# Editorial: Differential diagnosis of erythrocytosis and analysis of their clinical utility

**DOI:** 10.3389/fmed.2025.1742762

**Published:** 2025-12-11

**Authors:** Danijela Lekovic, Marta Sobas, Marko Lucijanic

**Affiliations:** 1 Faculty of Medicine, University of Belgrade, Belgrade, Serbia; 2 Clinic of Hematology, University Clinical center Serbia, Belgrade, Serbia; 3 Toruniu Collegium Medicum im Ludwika Rydygiera w Bydgoszczy, Uniwersytet Mikolaja Kopernika, Bydgoszcz, Poland; 4 Klinicka bolnica Dubrava, Zagreb, Croatia; 5 Medicinski Fakultet, Sveuciliste u Zagrebu, Zagreb, Croatia

**Keywords:** erythrocytosis, POEMS, TEMPI, differential diagnosis, erythropoeitin

Erythrocytosis is defined as an abnormally high red-cell mass (typically >125% of predicted) (1). In practice, elevated hemoglobin or hematocrit raises the concern, but true absolute erythrocytosis must be distinguished from relative hemoconcentration (e.g., dehydration, diuretics) (2). Once confirmed, erythrocytosis is classified along two axes: primary vs. secondary, and congenital vs. acquired (1, 3). Primary erythrocytosis arises from intrinsic defects of the bone marrow (e.g., *polycythemia vera* or congenital EPO-receptor mutations), whereas secondary erythrocytosis is driven by excess erythropoietin (EPO) in response to hypoxia or ectopic production (Borsani et al.). Both primary and secondary forms can be either inherited or acquired. Notably, up to half of seemingly “idiopathic” erythrocytosis cases still have no identifiable cause despite extensive workup (3).

In primary forms, EPO levels are low or normal due to feedback from high oxygen delivery (Borsani et al.). The classic example is PV (JAK2 V617F or exon 12 positive), but rare inherited primary forms also exist (e.g., heterozygous *EPOR* mutations) (3). In congenital primary erythrocytosis the defect lies within the EPO receptor or downstream signaling, and patients typically present with lifelong polycythemia without other causes. There are no formal guidelines for treating inherited primary erythrocytosis; many patients require only observation or phlebotomy for hyperviscosity symptoms (3).

Secondary erythrocytosis is far more heterogeneous, driven by external EPO stimulation. Chronic hypoxemia is a common trigger: lung diseases (chronic obstructive pulmonary disease, interstitial lung disease, or sleep apnea), high-altitude residence, and cyanotic heart disease can all raise EPO and RBC mass (Borsani et al.) (4). In fact, chronic cyanotic congenital heart defects (e.g., unrepaired Eisenmenger syndrome) produce a marked hypoxic drive and severe erythrocytosis as a compensatory mechanism (Borsani et al.). Smoking is another important cause: elevates carboxyhemoglobin, inducing tissue hypoxia and a reversible polycythemia that typically resolves after cessation (5). Anabolic steroids and other hormones (testosterone, for example) can also stimulate RBC production, as can certainly drugs like SGLT2 inhibitors (6).

Tumor-associated erythrocytosis is a classic secondary cause. Renal cell carcinoma, hepatocellular carcinoma, hemangioblastomas and uterine fibroids can secrete EPO or EPO-like factors (4). In these cases, the erythrocytosis is “inappropriate” (high EPO despite high hemoglobin), and imaging is indicated to find occult malignancy (4). Other endocrine or renal disorders (e.g., renal artery stenosis) may also elevate EPO. Patients with smoking, hypoxemia, or tumors have elevated or inappropriately normal EPO levels, distinguishing them from primary PV.

A notable but under-recognized cause is hemochromatosis. In hereditary iron overload, excess transferrin-bound and non-transferrin-bound iron saturates erythroid precursors, facilitating hematopoiesis (7). Case series have reported moderately increased hemoglobin and hematocrit in hemochromatosis, presumably from this iron-driven erythropoiesis (7). Clinicians should therefore remember iron overload as a potential contributor to unexplained secondary polycythemia.

Congenital erythrocytosis (inherited secondary forms) arises from germline mutations that affect oxygen sensing or hemoglobin function (3) (Borsani et al.). The classic congenital causes include high-affinity hemoglobin variants (globin gene mutations) and defects in the oxygen-sensing pathway: mutations in *VHL, EGLN1* (PHD2), or *EPAS1* (HIF2α) lead to increased EPO production despite normal oxygen levels (8). These patients have lifelong erythrocytosis but do not have the complications of myeloproliferation seen in PV. Workup for congenital causes is warranted in young patients or those with a family history: specialized testing (P50 oxygen dissociation, sequencing of EPOR, VHL, EGLN1, EPAS1, etc.) may reveal an inherited mutation (3) (Anzej Doma et al.). However, even with modern gene panels, a substantial fraction of congenital erythrocytosis remains genetically “idiopathic” (3) (Anzej Doma et al.).

Rare multisystem syndromes can also present with erythrocytosis, often misleading clinicians toward PV. POEMS syndrome (polyneuropathy, organomegaly, endocrinopathy, M-protein, skin changes) is a plasma-cell disorder in which erythrocytosis is a frequent laboratory finding (9). Similarly, TEMPI syndrome (telangiectasias, erythrocytosis, monoclonal gammopathy, perinephric fluid collections, intrapulmonary shunting) is characterized by profound secondary erythrocytosis with very high EPO levels (10). Both of these conditions produce elevated hemoglobin through inflammatory or paraneoplastic mechanisms. For example, elevated VEGF in POEMS may drive increased blood cell counts, and TEMPI patients are often misdiagnosed as having JAK2-negative PV until their monoclonal gammopathy is recognized (10). Awareness of these rare syndromes is important so that secondary causes are not overlooked when neuropathy or other non-hematologic features are present.

Evaluating erythrocytosis begins with confirming true RBC excess and excluding relative causes. Once absolute erythrocytosis is established, the first step is to measure arterial oxygen saturation and serum EPO. A low EPO level should raise suspicion for PV, prompting JAK2 mutation analysis. Conversely, an elevated EPO suggests secondary erythrocytosis; in such cases one evaluates for hypoxemia (lung function, sleep study, cardiac shunts) or hidden tumors (renal ultrasound, abdominal imaging). If a congenital cause is suspected (young patient, family history), specialized testing for oxygen-sensing pathway genes and hemoglobin variants is indicated (Anzej Doma et al.). This tiered approach ensures accurate classification before instituting therapy.

This Research Topic includes five reports that illustrate these principles. Borsani et al. present an adult with unrepaired Eisenmenger physiology, in whom chronic hypoxemia–driven erythrocytos was initially alarming; their case highlights that the high hematocrit in this setting is compensatory and should not be overtreated (phlebotomy is reserved for hyperviscosity symptoms) (Borsani et al.). Anzej Doma et al. report an original series of 40 “idiopathic” erythrocytosis patients investigated by next-generation sequencing. After excluding PV and obvious causes, they found pathogenic or candidate variants (e.g., in *EGLN1*) in only a small minority, underscoring how rare true congenital mutations are and how most cases remain unexplained (Anzej Doma et al.). Two case reports focus on POEMS syndrome: Tan et al. describe an atypical POEMS case without the usual neuropathy, and Xu et al. report a POEMS patient whose predominant feature was refractory portal hypertension. Both illustrate how POEMS can masquerade as other diseases and remind clinicians to suspect it when erythrocytosis coexists with monoclonal gammopathy or systemic symptoms (Tan et al., Xu et al.). Finally, Bambo et al. provide a systematic meta-analysis of hematologic changes in diabetes, although not focused on polycythemia *per se*, their review emphasizes how common chronic conditions can subtly alter blood counts and potentially confound the assessment of erythrocytosis.

In summary, the differential diagnosis of erythrocytosis is broad. A methodical approach confirming true erythrocytosis, measuring EPO levels, and excluding PV by mutation analysis is essential. Clinicians must consider not only the usual causes (primary PV, lung disease, tumors, smoking) but also less common etiologies like hemochromatosis, congenital high-affinity hemoglobin, and rare plasma cell syndromes (POEMS, TEMPI). The contributions in this Research Topic exemplify these scenarios and provide guidance: from genetic testing in so-called idiopathic cases to careful case-based reasoning in complex presentations. Together, they underscore that unraveling the cause of erythrocytosis is critical for choosing the right management and avoiding inappropriate treatment.
